# Multicentric Carpo-Tarsal Osteolysis

**DOI:** 10.5334/jbsr.3018

**Published:** 2023-02-02

**Authors:** Lea Tannouri, Paolo Simoni

**Affiliations:** 1 ULB, BE; 2 University Children’s Hospital Buxelles, BE

**Keywords:** JIA, Children, Joints

## Abstract

MCTO is a rare autosomal-dominant inherited disorder that causes bone destruction and deformity of the appendicular bones in children, with the carpal and tarsal bones severely affected. Imaging can narrow the differential diagnosis between MTCO and other diseases that cause osteolysis of the joints in children. The MCTO diagnosis is confirmed by a mutation of the MAFB gene.

**Teaching Point:**

Multicentric carpo-tarsal osteolysis (MTCO) in childhood must be considered in the differential diagnosis of severe osteolysis on radiographs.

## Case Report

**Figure 1:**
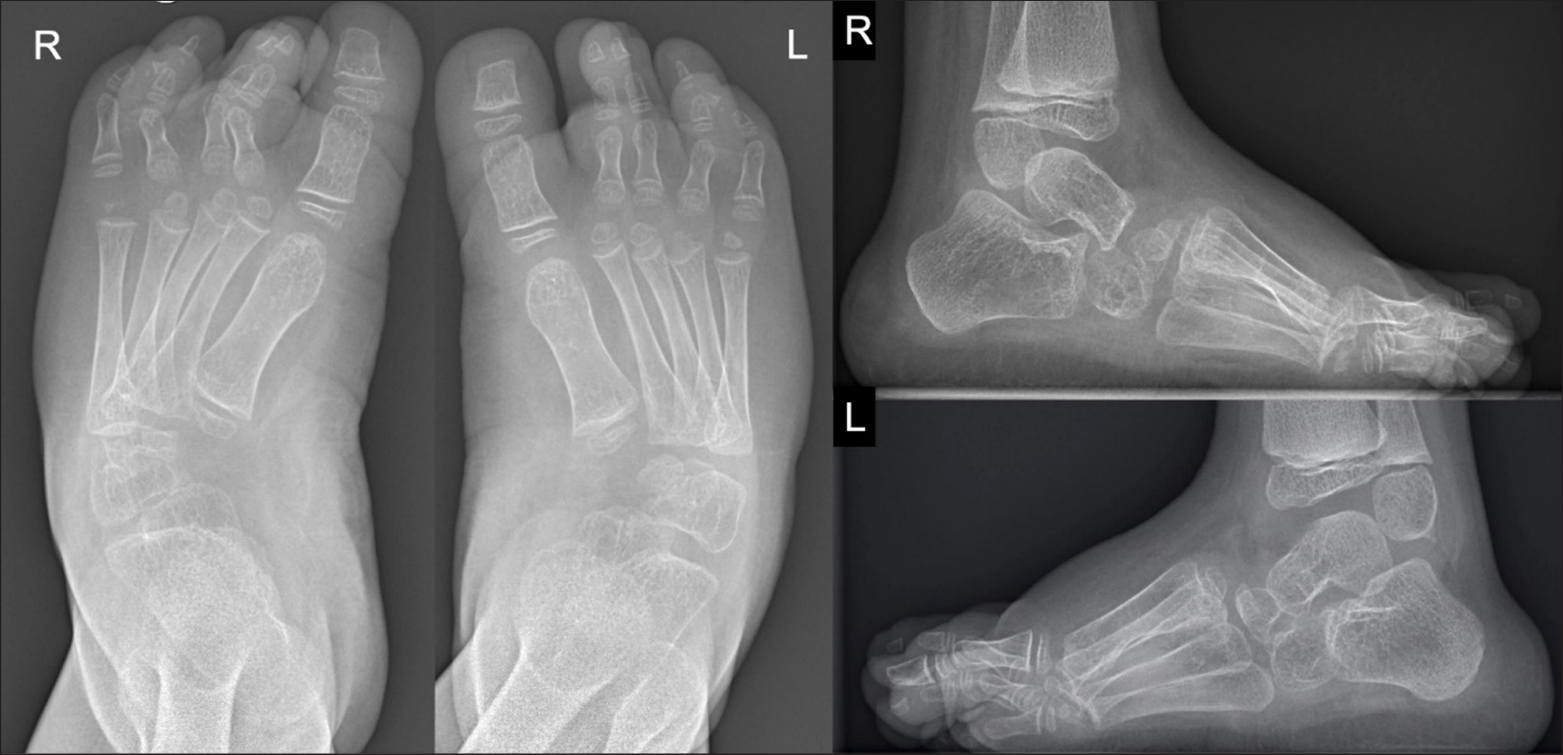
The radiographs of the ankle and feet reveal an extensive osteolysis of the tarsal bone, especially at the medial side of the midfoot and proximal metatarsal bone, causing a slight inward deformity of the forefeet.

**Figure 2:**
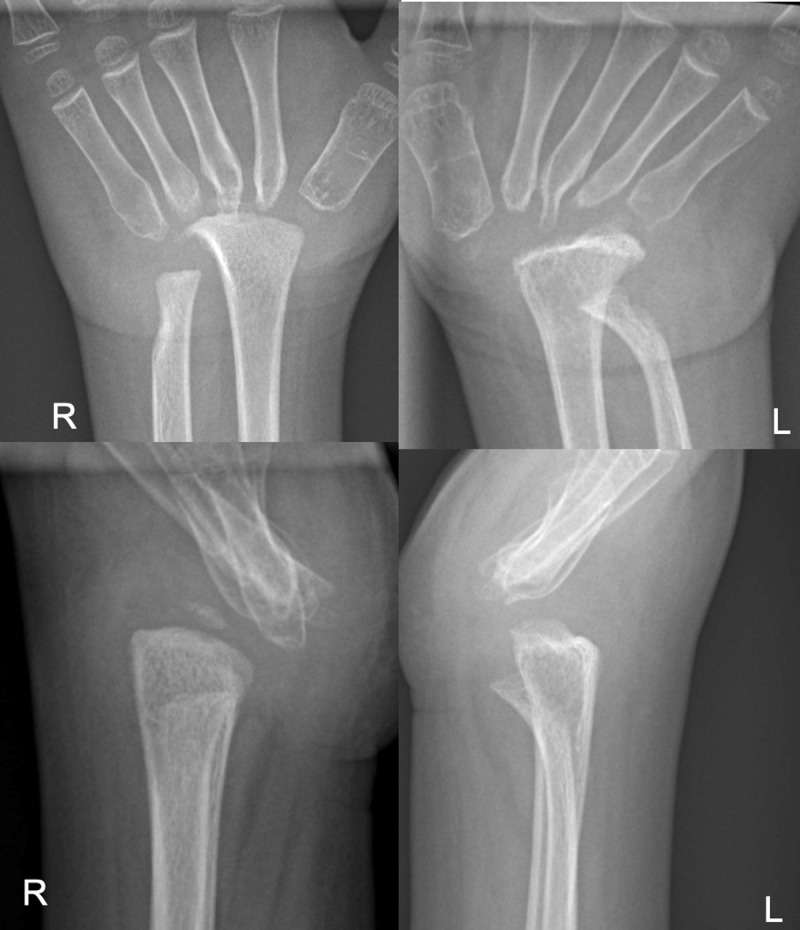
The radiographs of the hands, carpi and distal forearms show a massive osteolysis of the carpal bones bilaterally. The osteolysis also involves the proximal metacarpal bones, which appear tapered and anteriorly subluxated, especially at the right hand. Periarticular soft tissues are swollen with decreased transparancy. The osteolysis of the distal forearm results in radial epiphysis disappearance and radial and ulnar deformity on the left side.

A four-year-old girl was referred because her elbows, wrists and feet had been swollen and slightly painful for several months. An X-ray examination of the elbows, wrists and feet revealed extensive osteolysis of the carpal and tarsal bones and distal forearms, including the radial epiphysis of the carpal and metacarpal bones (Figures 1 and 2).

**Figure 3:**
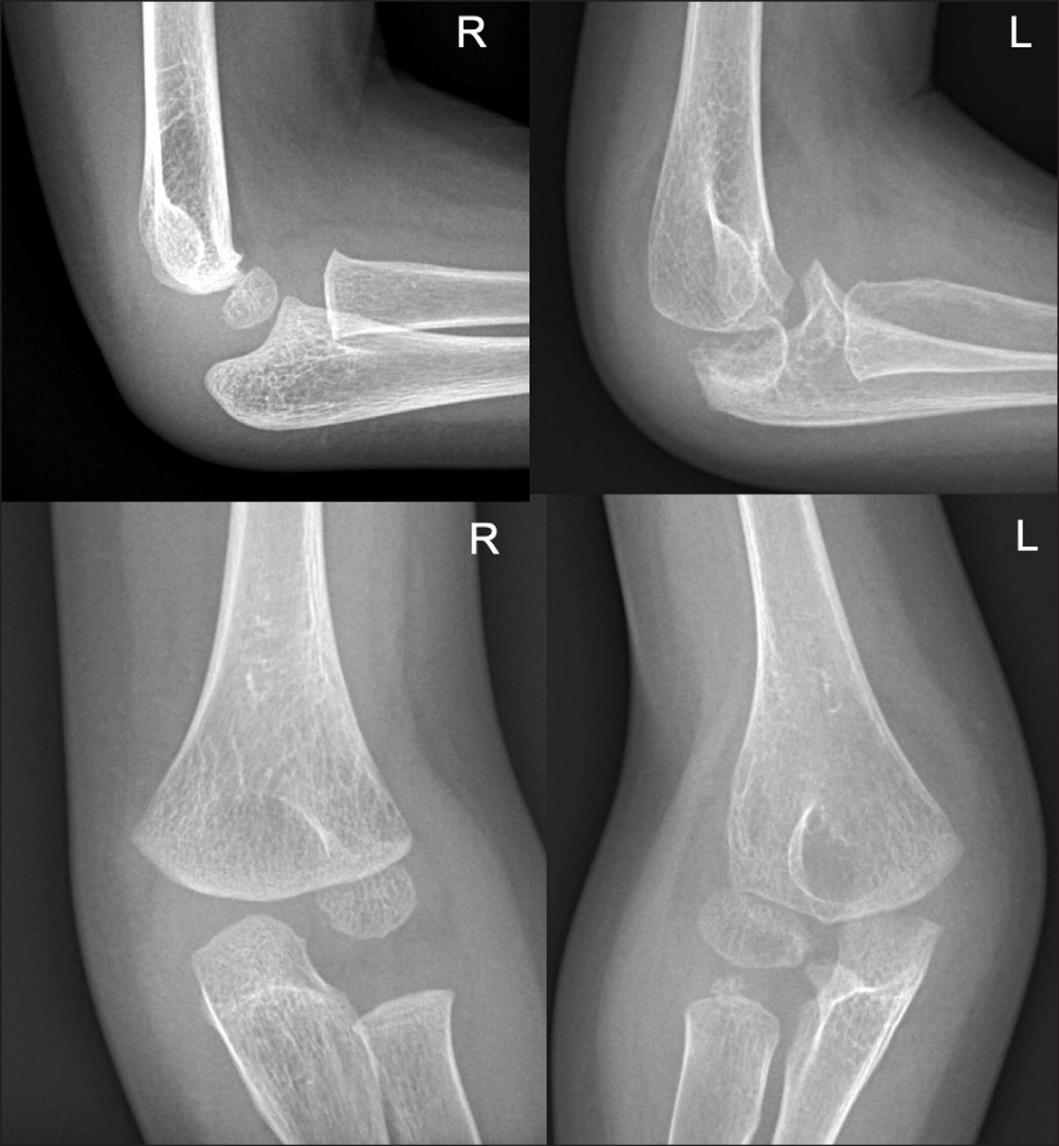
The radiographs of elbows show a significant osteolysis of the left elbow, leading to joint collapse. Soft tissues around the right elbow are swollen. The right elbow appears normal.

Extensive osteolysis of the left elbow was also visible (Figure 3). These osseous alterations were accompanied by abarticular soft tissues swelling of the left elbow (Figure 3). Some degree of tarsal and carpal subluxation was observed, as well as bony deformity of the ulna bilaterally (Figures 1, 2, 3).

Clinical evaluation and laboratory tests were not suggestive of autoimmune pathology. A genetic test revealed a de novo mutation of the MAFB gene (p.Ser70Leu). Based on this finding and the clinical findings, the diagnosis of multicentric carpo-tarsal osteolysis (MCTO) was made. As there is no specific treatment for MTCO, the girl was referred for regular follow-up for pain management and physiotherapy.

## Comments

MCTO is a rare autosomal dominant disorder. Similar de novo cases have also been reported [1]. MCTO is caused by a mutation of the MAFB gene (V-Maf Musculoaponeurotic Fibrosarcoma Oncogenic Homolog B) [1]. In MTCO, reduced MAFB gene expression increases osteoclastic activity, leading to massive bone resorption. As MAFB protein is also important for glomerular podocyte differentiation [2], its mutation can lead to progressive renal failure with proteinuria [2]. Our patient had mild proteinuria without renal failure. Patients with MCTO may have multiple craniofacial deformities. Some patients also have corneal opacities and cognitive impairment [2]. On radiographs, MCTO causes bone resorption in the appendicular joints. The carpal and tarsal joints are particularly affected [2]. Severe scoliosis may complicate MTCO [2]. Imaging helps to narrow the differential diagnosis between MTCO and other conditions, such as juvenile idiopathic arthritis (JIA), mucopolysaccharidoses, multicentric osteolysis nodulosis and arthropathy (MONA), juvenile hyaline fibromatosis syndrome and Winchester syndrome [2]. Although the diagnosis of MCTO can be suspected based on the preferential involvement of the carpus and tarsus on radiographs, as in our case (Figures 1, 2, 3), genetic testing to demonstrate a mutation of the MAFB gene is mandatory to confirm the diagnosis [1,4]. In contrast to MTCO, JIA causes chondrolysis, bone erosions, epiphyseal overgrowth, periarticular soft tissue swelling and joint deformities [5]. Mucopolysaccharidosis causes spherical vertebrae, short claw-like metatarsals, and pes cavus. Multicentric osteolysis nodulosis and arthropathy (MONA) is characterized by diffuse osteopenia and thinning of the bone cortex, especially in the metacarpals [6]. In Winchester syndrome, soft tissue swelling, osteopenia and cortical thinning are typically bilateral and symmetrical [7]. In hyaline fibromatosis syndrome, bone resorption is often observed in the distal phalanges, skull and long bones [8].

In MCTO, joint pain and soft tissue swelling gradually resolve during childhood, but joint stiffness and limited mobility are common disabling sequelae [2]. Treatment for MCTO includes supportive therapy and physiotherapy [6]. In selected cases, surgery may be considered [3].
